# A Study on User-Oriented Subjects of Child Abuse on Wikipedia: Temporal Analysis of Wikipedia History Versions and Traffic Data

**DOI:** 10.2196/43901

**Published:** 2023-07-17

**Authors:** Yanyan Wang, Jin Zhang

**Affiliations:** 1 School of Information Resource Management Renmin University of China Beijing China; 2 Research Center for Digital Humanities of RUC Renmin University of China Beijing China; 3 School of Information Studies University of Wisconsin-Milwaukee Milwaukee, WI United States

**Keywords:** child abuse, user-oriented subject, subject schema, subject change, popularity trend, temporal analysis, Wikipedia

## Abstract

**Background:**

Many people turn to online open encyclopedias such as Wikipedia to seek knowledge about child abuse. However, the information available on this website is often disorganized and incomplete.

**Objective:**

The aim of this study is to analyze Wikipedia’s coverage of child abuse and provide a more accessible way for users to browse child abuse–related content. The study explored the main themes and subjects related to child abuse on Wikipedia and proposed a multilayer user-oriented subject schema from the general users’ perspective.

**Methods:**

The knowledge of child abuse on Wikipedia is presented in the child abuse–related articles on it. The study analyzed child abuse–related articles on Wikipedia, examining their history versions and yearly page views data to reveal the evolution of content and popularity. The themes and subjects were identified from the articles’ text using the open coding, self-organizing map, and n-gram approaches. The subjects in different periods were compared to reveal changes in content.

**Results:**

This study collected and investigated 241 associated Wikipedia articles and their history versions and traffic data. Four facets were identified: (1) *maltreatment behavior* (n=118, 48.9%); (2) *people and environment* (n=28, 11.6%); (3) *problems and risks* (n=33, 13.7%); and (4) *protection and support* (n=62, 25.7%). A total of 8 themes and 51 subjects were generated from the text, and a user-oriented subject schema linking the facets, themes, subjects, and articles was created. *Maltreatment behavior* (number of total views = 1.15 × 10^8^) was the most popular facet viewed by users, while *people and environment* (number of total views = 2.42 × 10^7^) was the least popular. The popularity of child abuse increased from 2010 to 2014 but decreased after that.

**Conclusions:**

The user-oriented subject schema provides an easier way for users to seek information and learn about child abuse. The knowledge of child abuse on Wikipedia covers the harms done to children, the problems caused by child abuse, the protection of children, and the people involved in child abuse. However, there was an inconsistency between the interests of general users and Wikipedia editors, and the child abuse knowledge on Wikipedia was found to be deficient, lacking content about typical child abuse types. To meet users’ needs, health information creators need to generate more information to fill the knowledge gap.

## Introduction

### Background

As Roe versus Wade was overturned, the general public once again shifted their attention to reproductive rights and the right to health, and the discussion on women’s rights and health rose accordingly. Among various health-related problems women are facing, child abuse is a typical maltreatment that is widespread and hidden [1,2]. According to the UN Women [3], about 58% of women and girls killed in 2020 were perpetrated by intimate partners or other family members. Pan et al [4] reviewed 48 child sexual abuse (CSA) articles and found that the pooled overall rate for women who faced CSA was 24%.

Although girls are at more risk of sexual abuse, the risks of mental and physical abuses that boys face are equal as girls according to the World Health Organization (WHO). The Centers for Disease Control and Prevention reported that about 1 billion children experience abuse every year [5]. In the United States, the national rounded number of children exposed to abuse for the federal fiscal year 2020 was 618,000 [6]. And the children exposed to abuse usually experience more than 1 form of violence. For instance, in Tanzania, among adolescents aged 13-24 years who have experienced sexual abuse during childhood, more than 80% have also experienced physical violence [5].

Child abuse has serious impacts, including physical and mental harms and social problems (eg, homelessness and crime), on both females and males [2,7-9]. The study by Walsh et al [9] proved that child physical abuse, either alone or in combination with sexual abuse, increased chronic pain ratings among women. Exposure to child abuse was also associated with adult obesity among California women and elevated inflammation among midlife women [10,11]. Furthermore, CSA was one of the reasons for the repeated abuse in adolescence, with repeated abuse in adolescence associated with perpetration of violence [12,13].

### Child Abuse Online Education

Lack of awareness about child abuse can lead to instances of child abuse. The WHO has declared that building awareness and teaching skills for identifying child abuse are effective measures [14]. The Child Molestation Research and Prevention Institute has also reported that 95% of sexual abuse can be prevented through education and awareness [15]. Therefore, it is necessary to educate children, health care and social service providers, and the general public about child abuse.

With the development of information and communication technology and the internet, online education interventions provide opportunities for different groups to receive child abuse training and gain related knowledge [16]. The outbreak of COVID-19 has reiterated the importance of child abuse online education from different perspectives. Economic and caregiver-related stresses caused by the COVID-19 pandemic have led to an increase in child abuse [17,18]. Additionally, because people should isolate if they receive a positive COVID-19 test result, they need online health information and health care services.

Online child abuse education resources and programs have been developed for the general public. McMaster University made the VEGA educational resources publicly available online in 2020 to provide foundational knowledge of child abuse and improve skills for identifying and responding to child abuse [19]. The Social Connectivity Online Prevention and Experience website focuses on raising awareness among children and young adults about online pornography and sharing of explicit imagery [20]. Videos and online presentations about child abuse are also available for children [21,22]. Game playing is another popular and effective way to educate children about safety [22,23].

For health care and social service providers, online education is more effective than traditional learning approaches [24,25]. Health professionals and students also receive online child abuse education, such as online courses and trainings [26,27].

### Wikipedia as a Health Education Resource

Wikipedia is a free online encyclopedia generated collaboratively and continuously by Wikipedia editors. The goal of this online encyclopedia is to encompass “the sum of all human knowledge” and fill gaps in knowledge, making it a frequently used electronic knowledge resource [28,29]. When searching clinical words, Wikipedia pages are often ranked higher than pages from other websites, making it an easily accessible source of knowledge for general users [30]. It is also recognized as one of the most popular websites providing health information and one of the leading resources for health professionals and medical professionals [31]. According to Wikimedia Statistics, Wikipedia articles were accessed by 2 billion unique devices monthly and edited 176 million times during the past year [32].

However, the crowdsourced material on Wikipedia is anonymous and sometimes not contributed by experts, leading to doubts about the quality and credibility of the information. Hunter et al [33] compared Wikipedia with Lexicomp (Wolters Kluwer Health) and discovered that the medication information on Wikipedia was not always comprehensive. By contrast, Park et al [34] found that although some health care providers thought that using Wikipedia should be monitored, others trusted Wikipedia as a reliable information source. Rechenberg et al [35] evaluated 211 Wikipedia medical articles and found that 42.5% of the investigated articles’ quality was high enough to be used in medical examinations and clinical work. Other studies also verified that the quality of Wikipedia medical content was consistently high [36,37]. As the quality and reliability of Wikipedia articles are now being recognized by an increasing number of scholars, the use of Wikipedia in education has increased. Researchers have also proposed frameworks and strategies for incorporating Wikipedia into education to overcome its drawbacks [38]. They also trained students in editing Wikipedia content and improved students’ skills in information quality assessment [38,39].

### Goal of This Study

The use of Wikipedia as a source of health and medical information is limited by the lack of organization in its content. Although it is a popular knowledge resource, the information on Wikipedia is contained in individual articles and linked together via hyperlinks. This lack of a comprehensive directory makes it difficult for users to navigate and browse information effectively, particularly in the case of health and medical information.

To address this issue, a study was conducted to develop a subject schema of child abuse based on child abuse–related articles on Wikipedia. A subject schema is a hierarchy of subjects related to certain topics, which can be used to organize online information of certain topics on websites, digital libraries, information repositories, among others [40]. The user-oriented schema of child abuse provides a user’s perspective on the topic, making it easier for users to locate relevant information of child abuse. The subject schema expands the whole picture of child abuse–related knowledge on Wikipedia and identifies subjects related to child abuse, revealing relationships between subjects and articles. This type of subject schema can improve the effectiveness and efficiency of information seeking and knowledge learning on Wikipedia.

In addition to the subject schema, the study investigated the history versions of child abuse articles on Wikipedia to illustrate the evolution of child abuse–related subjects. The popularity of articles was also examined as an indicator of the general public’s interests in specific content [41]. This information can be used to help Wikipedia editors and health professionals generate more useful child abuse information and knowledge in need. By providing a user-oriented subject schema and investigating the evolution of child abuse knowledge on Wikipedia, this study aims to assist researchers in understanding the change of the child abuse topic over the time, and improve the organization and accessibility of health and medical information for the general public. The research questions (RQs) are as follows:

- RQ1: What is the internal evolution of the child abuse topic on Wikipedia?
  - RQ1a: What are the main themes and subjects of the topic?
  - RQ1b: What subjects are emerging and disappearing in the investigated period?
- RQ2: What is the external popularity trend of the child abuse topic on Wikipedia?

## Methods

### Overview

To demonstrate the internal and external evolutions of the child abuse topic, both the content and traffic data of child abuse–related articles from 2010 to 2017 on Wikipedia were collected and analyzed. The content of the articles reflects the internal characteristics and the page views data of the articles reflect the external popularity.

### Data Collection

#### Selection of Articles

The largest user-generated online encyclopedia, Wikipedia, was selected as the data source, because its content is generated by the general public and its history data are accessible [42,43]. Wikipedia is an online encyclopedia consisting of numerous articles linked to each other. To reveal the evolution of the child abuse knowledge on Wikipedia, we selected the articles related to child abuse in 2 ways and collected the history traffic data of these articles. The first way to seek relevant articles was a query search offered by Wikipedia. The terms “child abuse” and “child maltreatment” were used as search terms and the search results returned were examined by the researchers.

The second way was seeking the relevant articles via links. In the first way, we found the article “Child Abuse” on Wikipedia and regarded it as a starting point for seeking the associated articles. The articles in the “See also” section were relevant to the topic, called the first-level articles, while the articles contained in these articles’ “See also” sections were the second-level articles. When examining the third-level articles, they were either irrelevant or had been obtained in the previous steps. After seeking the articles by the 2 means, the researchers investigated the articles manually and selected the relevant ones.

#### History Data Collection

This study collected the data about the relevant articles from 2010 to 2017 and compared the content of the articles in different periods. The researchers regarded 2 years as a period, and thus, 4 periods were defined, which were 2010-2011 (period 1), 2012-2013 (period 2), 2014-2015 (period 3), and 2016-2017 (period 4). For each period, the last history version of each investigated article was collected by the WikipediR package run on R (R Foundation for Statistical Computing). The history versions were collected from the Wikipedia web pages. The “View history” page of an article displays its revision history and provides the link to every history version. This study compared the content of the last version (per versions history) of each article from every period to reveal the changes of the content.

Page views data of each article from 2010 to 2017 were obtained to reflect the external popularity of the topic. The page views data were extracted from Wikipedia data dumps that store all the historical page views data of all the Wikipedia articles [44].

### Data Analysis

#### Facet Identification

The associated articles obtained were grouped into several categories by the open coding method. As a Wikipedia article is usually about a specific type of object (eg, people, organization, event), the articles may be related to child abuse from different facets. To ensure the reliability of the coding results, 2 health professionals were invited to code the articles and the Cohen κ analysis was conducted. The coders identified and labeled the facets in terms of the articles related to them.

#### Theme and Subject Analysis

**Figure 1:**
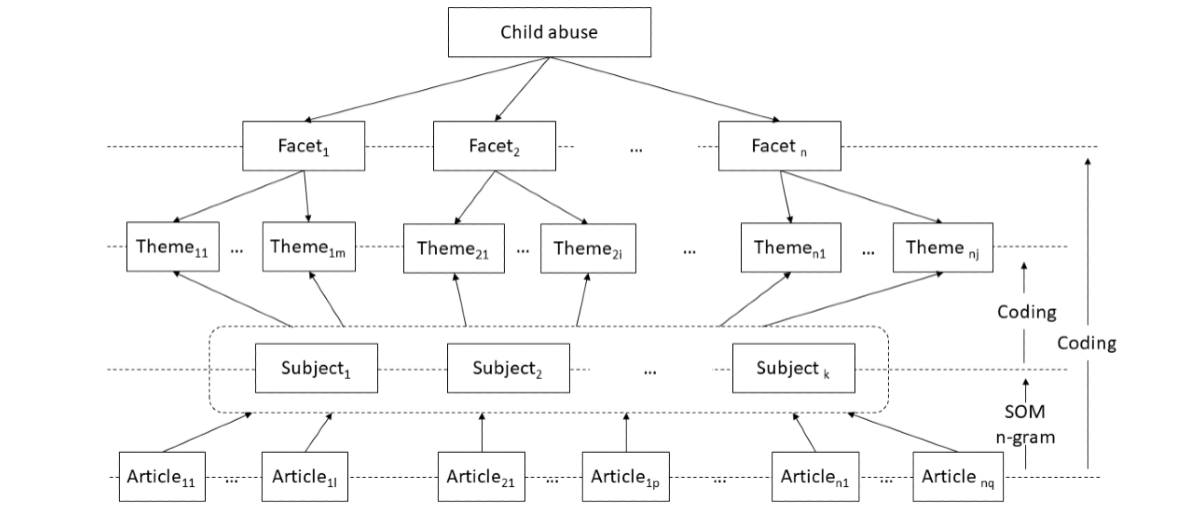
The relationships between the child abuse topic, facets, themes, subjects, and articles.

In addition to the facets, the subjects of the articles also illustrated the internal characteristics of the topic. In this study, a bottom-up strategy of subject identification was adopted. The researchers identified facets and categorized related articles into a category for each facet. The articles were then clustered into several groups using the self-organizing map (SOM) approach based on the similarity of their content. For each cluster, the researchers used the n-gram approach to extract high-frequency terms and phrases from the article text to identify the subjects. The researchers then manually generated themes for each facet based on the subjects identified. Figure 1 illustrates the structure of the facets, themes, subjects, and articles. This approach allowed for a comprehensive and user-oriented subject schema of child abuse on Wikipedia.

#### Text Data Processing and Cluster Generation

The high-frequency terms reflect the popular subjects of the child abuse topic from the Wikipedia editors’ perspective. However, some important subjects attracting less attentions cannot be recognized in this way, so the researchers clustered the 2017 versions of the articles related to each facet first by the SOM approach and then extracted the high-frequency terms for each cluster. The SOM approach is a widely used unsupervised learning neural network approach measuring and visualizing similarities among items [45,46]. In this study, we used the text data of the articles so that the SOM approach can measure the content similarities of the articles.

To cluster the articles, their text was first processed. The punctuations and stop words in the text were removed, and the terms were stemmed. The frequencies of all the terms in each article’s 2017 version were counted and summed, and the terms whose total frequencies less than 4 were removed. Then a term frequency-inverse document frequency (TF-IDF) matrix was generated for each facet. Its columns were the terms and its rows were the articles. Each value of the TF-IDF matrices (*w_ij_*) was calculated based on Equation 1.

*w_ij_* = *a_ij_* × log(*m*/*e_j_*) **(1)**

where *a_ij_* is the frequency of term *j* in the 2017 version of article *i*; *m* is the number of articles in the matrix; *e_j_* represents the number of articles containing the term *j* in the matrix; and *w_ij_* is the final value that refers to term *j* and article *i* in the matrix.

The TF-IDF matrices obtained were the input matrices for the SOM analysis and the output were the SOM displays. On the SOM displays, the articles were located in terms of the similarities among them and the articles more similar to each other were grouped into 1 cluster. The researchers grouped the articles into different clusters based on the locations of the articles on the SOM displays.

After the articles were clustered into specific clusters, the n-gram approach extracted the high-frequency 2-, 3-, and 4-word terms/phrases from the text of each cluster, and then the researchers examined the high-frequency terms/phrases to identify the popular subjects. The relationships between articles and subjects were many to many.

To reveal the internal evolution of the topic, the changes in the content, themes, and subjects from 1 period to the next were illustrated. For each facet, the latest version in each investigated period was collected and the frequencies of terms/phrases were calculated for each period. The terms/phrases that increased/decreased the most from 1 period to the next were obtained. How the theme and subject changed was observed from the increases and decreases of the term/phrases.

### Ethical Considerations

This study did not require approval by the local research ethics board because only publicly available data were used and this study involved no humans or human interaction.

## Results

### Reliability Tests and Facets

A total of 241 child abuse–related articles were collected on Wikipedia and the relevant articles were assigned to 4 facets by the open coding method. The Cohen κ value was 0.658 (*P*<.001), which means a substantial agreement was obtained between the 2 coders [47]. Table 1 lists the facet identified and the number of related articles, with the articles assigned to each facet presented in Multimedia Appendix 1. The *maltreatment behavior* facet was the most popular among the 4 facets and the *protection and support* facet ranked the second place. These findings reveal that, on Wikipedia, the knowledge about the maltreatment and prevention of child abuse was more than that about specific people, social environment, causes of child abuse, and problems caused by child abuse.

**Table 1:** Facets of child abuse and related concepts on Wikipedia (N=241).

Facet	Articles, n (%)	Description
*Maltreatment behavior* (F1)	118 (48.9)	Articles related to maltreatment of children, including mental and physical abuse, violence, harm, neglect, and subordination to children
*People and environment* (F2)	28 (11.6)	Articles related to specific persons or groups and the social environment they live in
*Problems and risks* (F3)	33 (13.7)	Articles related to health problems and risks, including the causes and consequences and effects of child abuse
*Protection and support* (F4)	62 (25.7)	Articles related to policies, laws, research studies, literary and artistic work, treatments, people, and organizations that support and protect children, and prevent children from being abused

### Articles Creation and Traffic Changes

Based on the creation time of the relevant articles, the articles created in the investigated 4 periods for each facet are listed in Multimedia Appendix 2. Table 2 presents the number of articles created from 2010 to 2017. The total number of articles created each year decreased from 2010 to 2017. The F1 facet had much more new articles than the other 3 facets during the investigated period, contributing to the growth of the child maltreatment–related content to a large extent. The F4 facet also played a relatively important role in the growth of the related articles compared with the remaining 2 facets.

**Figure 2:**
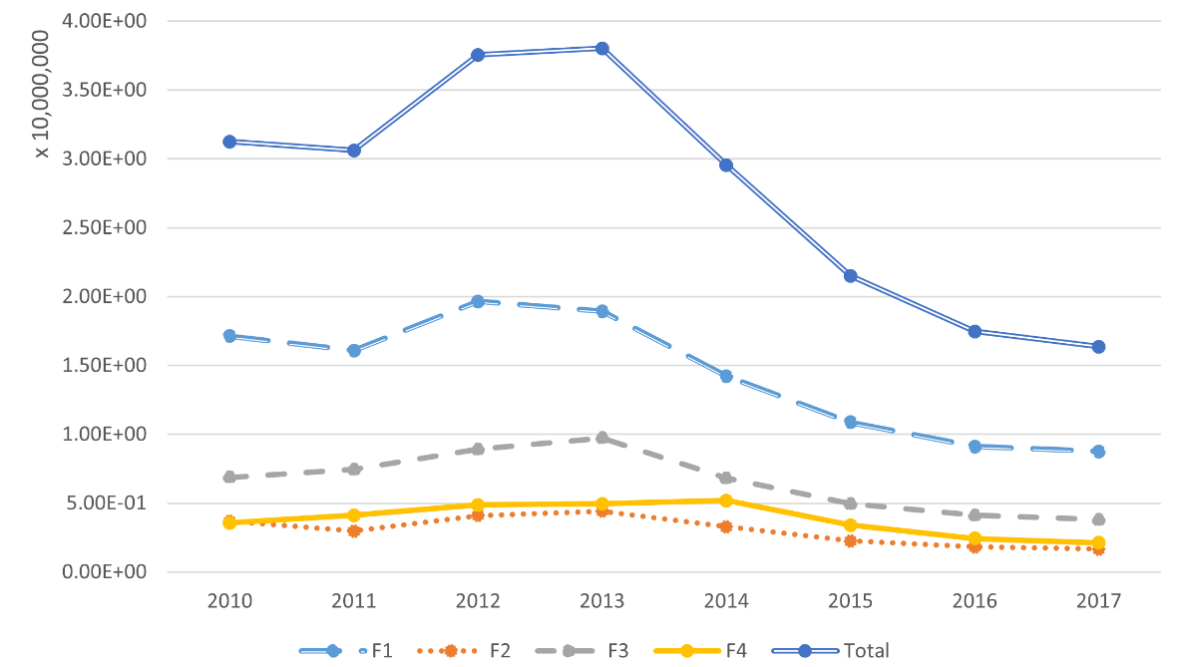
Numbers of yearly page views during 2010 to 2017.

The yearly page views data for each facet are displayed in Figure 2 and Table 3, which reveal that the F1 facet always had the highest yearly page views, while the F2 facet received the least page views among all the facets. The trend of page views of the child abuse topic increased from 2010 to 2013, but it decreased after 2013. It shows that the general users’ interests in child abuse kept decreasing after 2013, although the dropping became slow.

A comparison of the 4 facets shows that the popularities of the facets were consistent with the numbers of related articles, which indicates that the general users’ interests were consistent with the Wikipedia editors’ interests.

**Table 2:** Number of articles created between 2010 and 2017.

Facet	2010	2011	2012	2013	2014	2015	2016	2017
F1	7	7	4	6	5	4	6	2
F2	0	2	3	1	1	0	0	0
F3	3	1	0	1	0	1	0	1
F4	3	1	2	1	2	3	1	1
Total number of articles	13	11	9	9	8	8	7	4

**Table 3:** Numbers of yearly page views during 2010 to 2017.

Year	F1	F2	F3	F4	Total
2010	1.71 × 10^7^	3.68 × 10^6^	6.87 × 10^6^	3.57 × 10^6^	3.13 × 10^7^
2011	1.61 × 10^7^	2.96 × 10^6^	7.45 × 10^6^	4.13 × 10^6^	3.06 × 10^7^
2012	1.97 × 10^7^	4.11 × 10^6^	8.91 × 10^6^	4.88 × 10^6^	3.76 × 10^7^
2013	1.89 × 10^7^	4.42 × 10^6^	9.72 × 10^6^	4.96 × 10^6^	3.80 × 10^7^
2014	1.42 × 10^7^	3.30 × 10^6^	6.83 × 10^6^	5.20 × 10^6^	2.95 × 10^7^
2015	1.09 × 10^7^	2.26 × 10^6^	4.96 × 10^6^	3.41 × 10^6^	2.15 × 10^7^
2016	9.10 × 10^6^	1.81 × 10^6^	4.12 × 10^6^	2.43 × 10^6^	1.75 × 10^7^
2017	8.76 × 10^6^	1.67 × 10^6^	3.81 × 10^6^	2.13 × 10^6^	1.64 × 10^7^
Total	1.15 × 10^8^	2.42 × 10^7^	5.27 × 10^7^	3.07 × 10^7^	2.22 × 10^8^

### Themes and Subjects

#### Overview of Facets

Using the bottom-up strategy, we clustered articles related to each facet, with the results included in Figures S1-S4 in Multimedia Appendix 3. These figures show the clusters generated by the SOM approach for each facet, with the subjects identified based on the high-frequency terms and phrases extracted from the text of the clusters. Tables S1-S4 in Multimedia Appendix 3 list the clusters and their related subjects, as well as the high-frequency terms and phrases obtained for each cluster.

**Figure 3:**
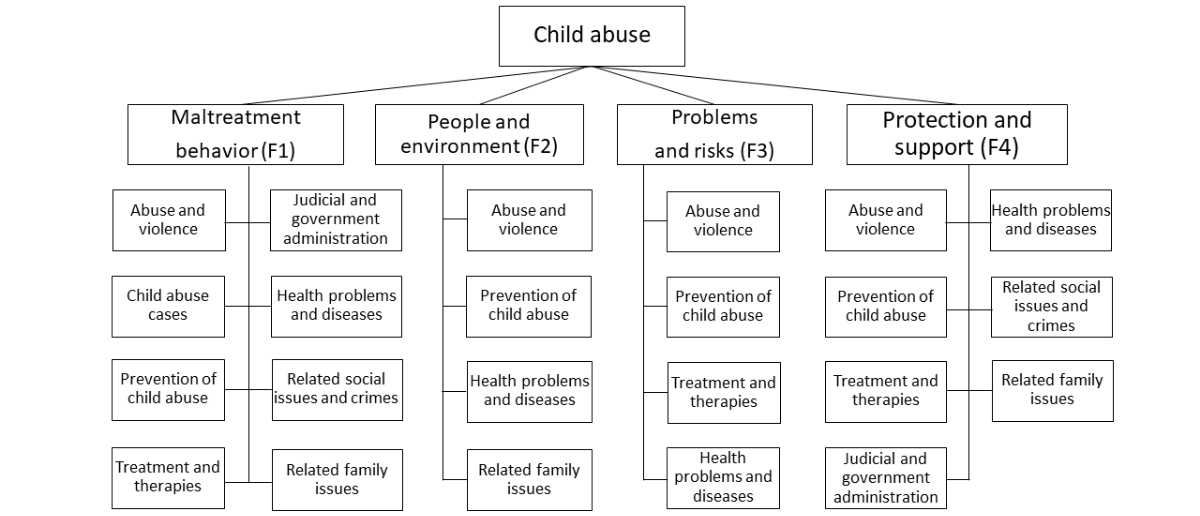
A part of the schema of the child abuse topic.

The researchers examined the subjects of the clusters for each facet and generated themes for each facet by manually coding them. The themes of each facet are listed in Figure 3 and the subjects in each theme are included in Multimedia Appendix 4. In total, 8 themes were identified in terms of the content of the articles, including *abuse and violence*, *child abuse cases*, *prevention of child abuse*, *treatment and therapies*, *judicial and government administration*, *health problems and diseases*, *related social issues and crimes*, and *related family issues*. Although the 4 facets are about different objects of child abuse, common themes and subjects were discovered for the different facets, revealing that categorizing articles based only on titles and the objects can omit relationships and similarities between the content of the articles. Therefore, discovering commonalities in the content of the articles and generating relationships between the articles based on these commonalities are necessary.

Multimedia Appendix 4 illustrates the relationships between the facets, themes, and subjects. Multimedia Appendices 3 and 4 clearly demonstrate the generation of the clusters, subjects, and themes of each facet step by step.

#### The Maltreatment Behavior Facet (F1)

Multimedia Appendices 3 and 4 show that the *abuse and violence* and *child abuse* cases themes appeared in most clusters of F1, indicating that they were the most popular themes among all the themes of F1 from the perspective of Wikipedia editors. The *abuse and violence* theme included several subjects, such as *sexual abuse, domestic violence, physical abuse, emotional abuse, neglect*. Among these different types of abuse and violence, *sexual abuse* and *domestic violence* occurred in more clusters than the other types, suggesting that knowledge about these 2 types of child maltreatment was more prevalent on Wikipedia than other types of child maltreatment. For instance, the story of Beth Thomas, who was sexually abused as a child, was mentioned in an associated article. Several articles were related to a series of Catholic sex abuse cases or scandals, such as the sexual abuse scandal in Fall River diocese. *Domestic violence* included both physical (eg, corporal punishment) and mental (eg, neglect) abuse to children. Famous domestic violence cases, such as the Collingswood Boys, were also demonstrated in the relevant articles of this theme. *Social issues* and *family issues* could be the potential cause of domestic violence.

*Health problems and diseases and prevention of child abuse* were 2 other popular themes of this facet. The former had related subjects such as *mental illness, physical illness,* and *reproduction*. Both physical and emotional abuse to children can cause health problems, such as stress disorder. *Reproduction* was also a typical subject of concern for some people exposed to domestic abuse. Wikipedia provided knowledge about reproductive health, family planning, and abortion. To improve health conditions in children, various organizations made their contributions, including nonprofit organizations, news and media, government departments, and law enforcement agencies. The WHO was one of the largest organizations helping developing child health globally. The police (eg, the South Yorkshire Police) and the news media (eg, BBC News and the Daily Telegraph) played an important role in protecting children and youth. Further, the criminal justice and laws were designed to hold criminals accountable and restore those subjected to abuse to their original situation as much as possible.

#### The People and Environment Facet (F2)

Same as F1, *abuse and violence*, *prevention of child abuse*, and *health problems and diseases* were the main themes of this facet, as shown in Multimedia Appendices 3 and 4. In addition, the *related family issues* theme was another focus of this facet. Subjects such as *immigrant family*, *nuclear family*, *family policy*, *family model*, and *marriage problem* were all associated with this theme. The *family issues* might be caused by government policies, laws, economy status, culture, and so on. The *family issues* would potentially cause violence and abuse and health problems.

There were also a number of terms/phrases associated with the psychology concept *attachment*. The attachment theory is about interpersonal relationships. At first, this theory only studied the context of child and parents, but in the past decades it was extended to adult relationships. Therefore, this concept was related to *mental illness*, a subject related to the *health problems and diseases* theme.

#### The Problems and Risks Facet (F3)

The most salient theme of the F3 facet was *health problems and diseases*, as it appeared in all 3 large clusters (Multimedia Appendices 3 and 4). This theme had *mental illness* and *physical illness* as its subjects, with many illnesses mentioned in the associated articles, including Geschwind syndrome, temporal lobe epilepsy, psychosomatic disorders, conduct disorder, among others. Some articles were on the causes of *health problems*, such as narcissistic parents and narcissistic parenting affecting children’s behaviors and attitudes.

The other primary themes were *treatment and therapies*. Similar to the *health problems and diseases* theme, a series of related *therapies* were contained in the Wikipedia articles, such as the Karpman drama triangle, attachment therapy, dialectical behavior therapy, multisystemic therapy, and so forth.

#### The Protection and Support Facet (F4)

In addition to *abuse and violence* and *prevention of child abuse*, the F4 facet had *judicial and government administration* and *related social issues and crimes* as its salient themes. Regarding the *judicial and government administration* theme, the *laws* of child and youth protection were one of the focuses of the Wikipedia editors. Some laws were enacted for children, such as the Child Abuse Prevention and Treatment Act, while some were not for specific groups, such as the Sexual Offences Act. The organizations, technologies, news reports, and research also attracted the Wikipedia editors’ attentions.

Considering the protection of children and youth, different countries had various *organizations of child and youth protection*. For instance, there is an Irish charity (Irish Society for the Prevention of Cruelty to Children) that protected children and in the United States the Supreme Court of the United States also helped protect children and youth. Some organizations such as the Internet Watch Foundation provided technologies and information that contributed to child protection. The Cleanfeed content blocking system used the child abuse image content URL list offered by the Internet Watch Foundation to block child pornography. News media such as the Guardian also made contributions to child and youth protection by *news reporting*. For the *research on child abuse*, the child abuse investigation team in the United Kingdom was responsible for investigating child abuse and offenses related to minors.

### Changes of Themes and Subjects

#### Internal Evolution of the Child Abuse Topic

To explore the internal evolution of the child abuse topic, the emerging and disappearing themes and subjects in the topic from 1 period to another were demonstrated. Multimedia Appendix 5 includes the terms/phrases whose frequencies increased or decreased the most from 1 period to the next for the 4 facets, respectively (Tables S1-S4 in Multimedia Appendix 5). The themes relevant to the terms/phrases were identified by the researchers based on the themes and subjects generated in Multimedia Appendices 3 and 4. After reviewing the changes of the terms/phrases and themes in Multimedia Appendix 5, Table 4 summarizes their growing and diminishing patterns.

New subjects were found regarding the changes in the frequencies of terms/phrases, which were *man protection* and *woman protection*. These subjects were more relevant to adults, indicating that the protection of adults would contribute to the protection of children and youth, and people caring about children also paid attention to the harms done to adults. The *man protection* subject rose only from periods 3 to 4. It implies that the Wikipedia editors’ interests not only focused on children and youth’s rights and protection, but also extended to other groups in recent years. However, the *woman protection* subject diminished, which was caused by the decrease in the discussion around maternity leave and working mothers.

**Table 4:** Growing and diminishing themes and subjects.

Pattern	Themes and subjects
Growing pattern^a^	Abuse and violence, prevention of child abuse (children and youth protection organization), health problems and diseases (reproduction), related social issues and crimes (man protection)
Diminishing pattern^a^	Related social issues and crimes (woman protection)

^a^The growing pattern refers to the themes and subjects whose associated terms and phrases kept increasing, while the diminishing pattern was on the opposite side.

#### The Maltreatment Behavior Facet (F1)

Table S1 in Multimedia Appendix 5 demonstrates that although the frequencies of some terms/phrases (eg, Catholic sexual abuse scandal and corporal punishment) about *abuse and violence* decreased in specific periods, the total frequency of the related terms/phrases increased rapidly during the investigated periods in F1. Among all the subjects of *abuse and violence*, the total frequencies of the terms/phrases related to *sexual abuse* and *child abuse* increased in all the 4 periods. The total frequency of *prevention of* *child abuse* rose from periods 1 to 4. The BBC News appeared in all the 3 comparisons, which means that it played an increasingly important role in children and youth protection.

#### The People and Environment Facet (F2)

In Table S2 in Multimedia Appendix 5, the total frequency of the terms/phrases about *related* *family issues* kept increasing from periods 1 to 4 in F2. Comparing periods 1 and 2, 3 of the frequency increasing terms were related to attachment theory. As mentioned before, there were 3 articles about this theory created in the second period. The increases in the attachment theory–related terms were caused by the generation of the corresponding articles.

An interesting finding was that the frequencies of the terms about *inequalities* and *discrimination* increased slightly from periods 2 to 3 but decreased slightly from periods 3 to 4. The discussion about gender equality, gender roles, and equal rights rose from periods 2 to 3. The discussion about maternity leave and working mothers reduced from periods 3 to 4. By contrast, the discussion about father’s rights grew from periods 3 to 4.

#### The Problems and Risks Facet (F3)

For the data on F3 in Table S3 in Multimedia Appendix 5, the total frequency of terms related to *health problems and diseases* increased in the 4 periods, which implies that the Wikipedia editors’ interests in these themes grew from 2010 to 2017. However, a particular finding shows that the term frequency of Disease Control and Prevention decreased rapidly from periods 1 to 2.

#### The Protection and Support Facet (F4)

The total frequencies of the *abuse and violence*, *prevention of* *child abuse*, and *judicial and government administration* themes in Table S4 in Multimedia Appendix 5 all increased during the investigated periods for F4. It reveals that the Wikipedia editors paid increasing attention to these 3 themes from periods 1 to 4.

For the *prevention of* *child abuse* theme, many of the relevant frequency increasing terms/phrases were about the *organizations* and for the *judicial and government administration* theme, many terms/phrases were about the *laws* of children and youth protection, which implies that these *organizations* and *laws* played an increasingly important role in children and youth protection. Moreover, there were 2 phrases, “Amber Alert” and “alert system,” referring to the technologies that helped protect children and youth. It reveals that the Wikipedia editors’ interests in the protection of children and youth increased from periods 1 to 4.

### User-Oriented Subject Schema Generation

Based on all of the facets, themes, and subjects, we generated a subject schema from the general users’ perspective, which is included in Multimedia Appendix 6. This subject schema connects facets, themes, and subjects to provide a comprehensive view of child abuse knowledge on Wikipedia. Using this schema, users can seek knowledge about child abuse from the facet level to the article level. If a user wants to seek relevant knowledge about a specific article or subject, they can access a higher-level subject or theme to quickly and easily find a series of associated articles or subjects.

## Discussion

### Principal Findings

This study investigated child abuse articles on Wikipedia and analyzed the topic’s evolution from 2010 to 2017. A total of 241 articles were analyzed, resulting in the identification of 4 facets, 8 themes, and 51 subjects. The articles covered the harms of child abuse, the problems caused by it, the protection of children, and the people involved in child abuse.

A user-oriented subject schema was established based on the facets, themes, and subjects discovered from the Wikipedia articles related to child abuse. Multimedia Appendix 6 demonstrates the subject schema of child abuse. This subject schema illustrates the coverage of child abuse knowledge on Wikipedia and shows the main concerns of child abuse from the users’ perspective. To generate the multilayer subject schema, this study proposed a bottom-up strategy to identify the items of different layers of the schema. The strategy used a mixed research methods approach, including the manually coding, SOM, and n-gram approaches. The strategy can be adopted to generate subject schemas of other topics.

Of the 4 facets, F1 had the most articles and page views, indicating that both editors and users paid the most attention to the harms and maltreatment. F2 had the least articles and page views. F3 had fewer articles than F4 but received more page views, suggesting inconsistency between editors’ and users’ interests. Overall, page views of child abuse increased from 2010 to 2014 but decreased afterward, indicating decreasing interest among general users.

Over time, child abuse subjects became increasingly diverse, but there were still gaps in knowledge. Some types of abuse, such as medical neglect, were not introduced, and there was a time lag between new theories and their incorporation into Wikipedia content. Health professionals and information creators should improve the comprehensiveness of health knowledge and generate new knowledge more quickly on online encyclopedias.

The study found a decrease in content related to the protection of women and an increase in content related to the protection of men among the analyzed articles. This suggests that the volume of knowledge of these 2 subjects changed in different patterns from 2010 to 2017. However, violence against women remains prevalent and has serious consequences, highlighting the need to raise awareness about the importance of protecting women among the health information creators.

### Interpretations and Comparison With Prior Work

#### Creation of New Articles

The articles created from 2010 to 2017 of F1 in Multimedia Appendix 2 show that those created during the first period mainly introduced different abuse types, such as narcissistic abuse, domestic violence, disability abuse, among others. During the second, third, and fourth periods, there were 2 types of new articles: (1) the articles about child abuse cases or scandals reported in the 3 periods or earlier (eg, the Kasur child sexual abuse scandal) and (2) the articles demonstrating the child abuse and child protection status in specific regions (eg, Child abuse in New Zealand). The remaining articles referred to particular abusive behaviors (eg, isolation to facilitate abuse) or child abuse types (eg, athletes and domestic violence).

Regarding F2, 4 new articles were created in the second period, much more than the other 3 periods. These 4 articles all concentrated on attachment theory and family-related problems. The research of attachment theory was first introduced in 1960s and this theory has been developing for more than 50 years. The psychology of religion field adopted this theory in 1990 and subsequently its development expanded.

F3 had 4 new articles in the first period, and the remaining 3 periods only had 1 new article, respectively. The new articles in the first period were all relevant to the impacts of child abuse on children. The theories and knowledge included in these 4 new articles were all proposed much earlier than the time during which these articles were created.

The new articles created in F4 referred to research (eg, journals), organizations (eg, International Society for the Prevention of Child Abuse and Neglect), technologies (eg, child abuse image content list), works of arts (eg, list of songs about child abuse), and laws and policies (eg, Karly’s Law) that protected children from being abused, as well as the treatment (eg, multisystemic therapy) that aimed to help the chronically violent youth.

These findings imply that the generation of these articles was usually much later than the generation of the corresponding theories and related knowledge. Although user-generated content platforms such as Wikipedia contain a large volume of knowledge, the most recent health knowledge was not always posted and, accordingly, the knowledge obtained from these platforms was incomplete.

#### The Popularity Trends of Child Abuse

**Figure 4:**
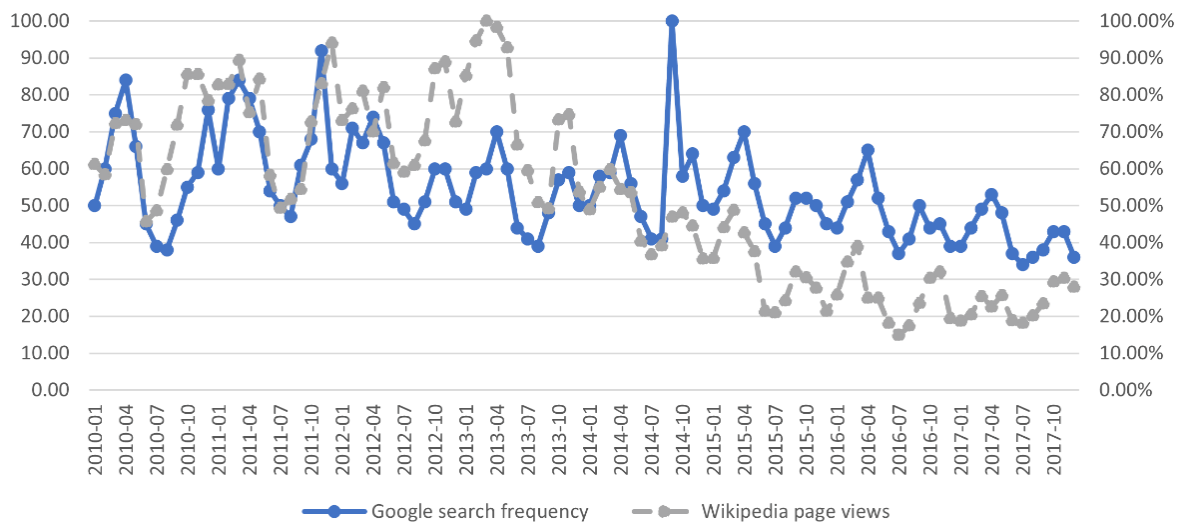
Trends of Google search frequency and Wikipedia page views of the Child Abuse article.

The findings illustrate that the external popularities of the child abuse topic declined from 2010 to 2017, although their content on Wikipedia kept increasing. Yoshida et al [48] found high correlations between Google search frequency and Wikipedia page views in terms of the data of personal name keywords. The researchers used child abuse as the search term to obtain Google search frequency data from Google Trends [49]. Figure 4 illustrates the trends of the Google search frequency and the Wikipedia page views from 2010 to 2017 for the child abuse topic. The x-axis represents the month from January 2010 to December 2017. The left y-axis represents the Google search frequency and the right y-axis is the ratio of the number of Wikipedia page views in a month to the highest number of monthly page views of child abuse, as the Google search frequency of a search term was the proportion of its number of searches at a time point to the highest number of searches over the selected period on Google [50].

The 2 curves in Figure 4 had similar shapes and the correlation coefficient (*r*) between the 2 indices was 0.608 (*P*<.001). Therefore, there was significant correlation between the search frequency and the page views on Wikipedia for child abuse, which confirms Yoshida et al’s results [48]. As the Google search frequency can reflect the public’s interests in the terms and the relevant concepts, the Wikipedia page views data also reveal the public’s interests [48].

As previously mentioned, F4 had more articles than F3, but F3 received more total page views, indicating that general users were more interested in the *problems and risks* associated with child abuse than in the *protection and support* of children. This misalignment between editors’ and users’ interests suggests the need to generate more knowledge about related problems and risks and to connect articles containing such content to the F3 facet to better meet users’ needs on Wikipedia.

#### Man Protection

The *man protection* subject started to grow from periods 3 to 4, especially the phrases “father’s rights” and “father’s rights movement” increasing fast during these periods. Examining the associated articles shows that the observed *man protection* subject mainly centered on father’s parental and reproductive rights.

The advocate of fatherhood emerged in the 1990s since fatherlessness became “the most critical social issue” [51]. The researchers claimed that many serious social problems (eg, violence, crime, unwed pregnancy) and health problems (eg, mental disorders) were strongly correlated with fatherlessness. A primary cause of fatherlessness was the gender bias in family law. The researchers examined the previous cases and proposed suggestions for protecting father’s rights to the government and eventually the father’s rights organizations (eg, Fathers 4 Justice) were built all over the world [51].

A relevant article of *man protection* was the “Father’s quota” article. Father’s quota is the paternity leave for fathers. This concept began to expand in the 1990s since it was introduced by the Norwegian welfare state. Studies about the paternity leave for fathers dated back to the early 1990s, and such studies remain active [52,53]. The researchers studied how fathers used the paternity leave, whether the paternity leave affected fathers’ work and income, the influences of fatherhood on children, and so forth [52-54]. The associated Wikipedia articles focused on the history and policies of father’s quota and paternity leave.

#### Woman Protection

According to Table 4, there was only 1 diminishing subject discovered in this study, which was the *woman protection* subject whose relevant terms/phrases decreased from periods 3 to 4 as a result of the diminishing discussion on maternity leave and working mothers.

Maternity leave was crucial for mothers and newborn, and children’s physical and mental health [55,56]. Longer maternity leave was associated with longer breastfeeding intentions [57,58]. It was also found that paid maternity leave benefited mothers and children’s mental health by decreasing postpartum maternal depression and intimate partner violence, and improving infant attachment and child development [56]. For the physical aspect, it helped decrease infant mortality and infant/mother rehospitalizations. As the paid maternity leave contributed to the increase in pediatric visit attendance and infant immunizations, it also lead to improvements in the physical health of infants and children.

However, not every country has enacted policies of paid maternity leave or other policies beneficial for parturient and newborn. For instance, the United States policies have not guaranteed paid maternity leave [55]. According to a survey in 2012, only 59% of US workers were eligible for 12 weeks of unpaid family and medical leave and in 2018 only 16% of all private industry employees were eligible for paid leave [59,60]. Furthermore, in recent years, a number of countries have decreased maternity leave (eg, Austria, Sweden, the Netherlands). In Austria, the maternity leave was 26 weeks in 1997, whereas it became 13 weeks in 2020. This phenomenon confirms the change of the *woman protection* subject data obtained from the Wikipedia history versions.

As mentioned before, currently in many parts of the world, woman’s rights have not been well protected, and a typical example is the maternity leave. An AllAfrica report [61] indicated that 12% of working women in South Africa are domestic workers who have difficulty receiving maternity leave or pay. Therefore, it is still necessary to call the general public’s attention to the inadequate protection for women.

### Implications

#### Implications of the Evolution Discovered

The internal evolution of the child abuse topic on Wikipedia helps health professionals and consumers, patients, and general users gain insights into the history and development of the child abuse knowledge from the general public’s perspective. The identification of 8 themes and 51 subjects in this study provides an overview of the child abuse knowledge covered on Wikipedia and demonstrates the extent of its coverage. The themes, subjects, and high-frequency terms/phrases identified, along with their relationships, can contribute to the development of health ontologies, consumer health vocabularies, subject headings, classifications, and thesaurus related to child abuse.

By identifying the themes and subjects, this study also revealed a potential knowledge gap among the general public regarding specific types of abuse, such as medical neglect, which were not covered in the analyzed articles. Health professionals can generate content to fill this gap and improve public awareness of abuse.

The study also found inconsistency between editors’ and users’ interests, as evidenced by the growth of content and external popularity trends for each facet. This knowledge can help health information creators and providers generate content that better meets users’ needs and increases traffic to the platform, so as to improve user loyalty for the platform.

#### Implications of the User-Oriented Subject Schema

This study established a multilayer user-oriented subject schema for child abuse knowledge based on the identified themes and subjects. Unlike the controlled vocabulary thesaurus generated by health professionals, such as MeSH (Medical Subject Headings), this schema was written in plain language and easily understandable by users. It provides a comprehensive overview of child abuse and enables users to gain insight into the topic.

System developers can use this schema as a basis for creating an online subject directory that provides navigation for general users seeking child abuse–related information. This will allow users to seek and acquire information more easily and efficiently than browsing web pages, and avoid getting lost in the vast amount of information available.

### Limitations and Conclusions

One limitation of this study is that only the data from 2010 to 2017 on Wikipedia were collected, so the findings cannot be generalized to all ages or platforms. Meanwhile, the “Gender bias on Wikipedia” article on Wikipedia claimed that the majority of Wikipedia editors were male, which might lead to a potential bias in the Wikipedia content. To obtain more generalized results, the data on child abuse generated from a longer period from more platforms will need to be collected and analyzed in future studies.

## Appendix

Multimedia Appendix 1 Articles collected of each facet.

Multimedia Appendix 2 Articles created in the investigated 4 periods for each facet.

Multimedia Appendix 3 SOM displays, clusters, high-frequency terms, and subjects of each facet.

Multimedia Appendix 4 Themes and subjects of each facet.

Multimedia Appendix 5 Terms whose frequencies increased or decreased the most from 1 period to the next and the themes of these terms of each facet.

Multimedia Appendix 6 The User-Oriented Subject Schema of Child Abuse on Wikipedia.

## Abbreviations

CSA: child sexual abuse
MeSH: Medical Subject Headings
RQ: research question
SOM: self-organizing map
TF-IDF: term frequency-inverse document frequency
WHO: World Health Organization

## Data Availability

The data sets generated or analyzed during this study are available from the corresponding author on reasonable request.
