# Supplementary material for: A Study on User-Oriented Subjects of Child Abuse on Wikipedia: Temporal Analysis of Wikipedia History Versions and Traffic Data
Source: J Med Internet Res. 2023 Jul 17;25:e43901. doi: 10.2196/43901 (PMC10390980; doi:10.2196/43901)
Supplement: Multimedia Appendix 1 [file jmir_v25i1e43901_app1.doc]

## **Articles collected of each facet**

| Facet | Articles |
| --- | --- |
| Maltreatment behavior: 118 articles | (1) 2009 Plymouth child abuse case, (2) A Modest Proposal, (3) Abortion, (4) Abuse, (5) Abusive power and control, (6) Adverse Childhood Experiences Study, (7) Athletes and domestic violence, (8) Aylesbury child sex abuse ring, (9) Baby farming, (10) Banbury child sex abuse ring, (11) Blackmail, (12) Brainwashing, (13) Bristol child sex abuse ring, (14) Candace Newmaker, (15) Catholic Church sexual abuse cases, (16) Child-on-child sexual abuse, (17) Child abduction, (18) Child abuse (skin signs), (19) Child abuse in China, (20) Child abuse in New Zealand, (21) Child abuse, (22) Child erotica, (23) Child grooming, (24) Child murder, (25) Child neglect, (26) Child of Rage, (27) Child pornography, (28) Child prostitution, (29) Child sacrifice, (30) Child sex tourism, (31) Child sexual abuse accommodation syndrome, (32) Child sexual abuse by UN peacekeepers, (33) Child sexual abuse in Australia, (34) Child sexual abuse in New York City religious institutions, (35) Child sexual abuse in Nigeria, (36) Child sexual abuse in the United Kingdom, (37) Child sexual abuse, (38) Cinderella effect, (39) Cleveland child abuse scandal, (40) Collingswood Boys, (41) Corporal punishment in the home, (42) Covert incest, (43) Cycle of violence, (44) Dave Pelzer, (45) Day-care sex-abuse hysteria, (46) Death of Baby P, (47) Death of Daniel Valerio, (48) Debate on the causes of clerical child abuse, (49) Derby child sex abuse ring, (50) Destabilisation, (51) Disability abuse, (52) Domestic violence, (53) Dysfunctional family, (54) Early infanticidal childrearing, (55) Exploitation of labour, (56) Extortion, (57) Female perversion, (58) Feral child, (59) Filicide, (60) Flying monkeys (psychology), (61) Franklin child prostitution ring allegations, (62) Haleigh Poutre, (63) Halifax child sex abuse ring, (64) Hostage, (65) Human trafficking, (66) Infant exposure, (67) Infanticide, (68) Institutional abuse, (69) Isolation to facilitate abuse, (70) Jimmy Savile sexual abuse scandal, (71) Jonathan Swift, (72) Kasur child sexual abuse scandal, (73) Keighley child sex abuse ring, (74) Kern County child abuse cases, (75) Kidnapping, (76) List of child abuse cases featuring long-term detention, (77) List of satanic ritual abuse allegations, (78) Margaret Garner, (79) Miyuki Ishikawa, (80) Mormon abuse cases, (81) Murder of Sylvia Likens, (82) Narcissistic abuse, (83) North Wales child abuse scandal, (84) Orkney child abuse scandal, (85) Outline of domestic violence, (86) Overlaying, (87) Oxford child sex abuse ring, (88) Parental alienation, (89) Penn State child sex abuse scandal, (90) Physical abuse, (91) Power and Control- Domestic Violence in America, (92) Psychological abuse, (93) Psychological manipulation, (94) Relationship between child pornography and child sexual abuse, (95) Religious abuse, (96) Rochdale child sex abuse ring, (97) Rotherham child sexual exploitation scandal, (98) Satanic ritual abuse, (99) School bullying, (100) School corporal punishment, (101) Sex-selective abortion, (102) Sexual abuse scandal in Fall River diocese, (103) Sexual abuse scandal in the Catholic archdiocese of Boston, (104) Sexual abuse scandal in the Catholic diocese of Orange, (105) Sexual abuse scandal in the Congregation of Christian Brothers, (106) Sexual abuse scandal in the English Benedictine Congregation, (107) Sexual abuse, (108) Sexual slavery, (109) Sibling abuse, (110) Slavery, (111) Social undermining, (112) Telford child sexual exploitation scandal, (113) The Cruel Mother, (114) Unfree labour, (115) USA Gymnastics sex abuse scandal, (116) Verbal abuse, (117) Victim playing, (118) Victimisation |
| People and environment: 28 articles | (1) Attachment in adults, (2) Attachment theory and psychology of religion, (3) Child soldiers in Sierra Leone, (4) Child, (5) Cinderella complex, (6) Enmeshment, (7) Extended family, (8) Family economics, (9) Family nexus, (10) Family, (11) Fathers’ rights movement, (12) Fathers as attachment figures, (13) Human bonding, (14) Hypergamy, (15) Inequality within immigrant families in the United States, (16) Juvenile delinquency, (17) Maternal bond, (18) Nuclear family, (19) Nurture kinship, (20) Parental abuse by children, (21) Paternity fraud, (22) Vocal school, (23) Rotten kid theorem, (24) Runaway (dependent), (25) School discipline, (26) Sociology of the family, (27) Teenage rebellion, (28) Work-family balance in the United States |
| Problems and risks: 33 entries | (1) Alcoholism in family systems, (2) Atlas personality, (3) Borderline personality disorder, (4) Child sexuality, (5) Complex post-traumatic stress disorder, (6) Conduct disorder, (7) Developmental impact of child neglect in early childhood, (8) Effects of domestic violence on children, (9) Emotional dysregulation, (10) Emotional self-regulation, (11) Enabling, (12) Externalizing disorders, (13) Foster care, (14) Healthy narcissism, (15) Infant mortality, (16) Karpman drama triangle, (17) List of countries by infant and under-five mortality rates, (18) Men’s health, (19) Narcissistic parent, (20) Oppositional defiant disorder, (21) Pedophilia, (22) Pseudobulbar affect, (23) Psychosomatic medicine, (24) Reactive attachment disorder, (25) Reduced affect display, (26) Self psychology, (27) Social determinants of health, (28) Spiritual crisis, (29) Substance abuse, (30) Sudden infant death syndrome, (31) Traumatic bonding, (32) Vulnerable adult, (33) Women’s health |
| Protection and support: 61 entries | (1) Abuse defense, (2) Abuse prevention program, (3) AMBER Alert, (4) Attachment-based psychotherapy, (5) Attachment in children, (6) Attachment parenting, (7) Attachment theory, (8) Attachment therapy, (9) Barnardo’s, (10) Bikers Against Child Abuse, (11) Campaigns against corporal punishment, (12) Child Abuse & Neglect, (13) Child abuse image content list, (14) Child abuse investigation team, (15) Child Abuse Prevention and Treatment Act, (16) Child Abuse Review, (17) Child Development Index, (18) Child development, (19) Child protection, (20) Child Protective Services, (21) Child sexual abuse laws in India, (22) Child sexual abuse laws in the United States, (23) Children’s rights, (24) Commission to Inquire into Child Abuse, (25) False allegation of child sexual abuse, (26) Family law, (27) Family therapy, (28) George Hosking, (29) Harry Stack Sullivan, (30) Identified patient, (31) Independent Inquiry into Child Sexual Abuse, (32) International Federation for Human Rights, (33) International Society for the Prevention of Child Abuse and Neglect, (34) Irish Society for the Prevention of Cruelty to Children, (35) Jehovah’s Witnesses’ handling of child sex abuse, (36) Jersey child abuse investigation 2008, (37) Journal of Child Sexual Abuse, (38) Karly’s Law, (39) Laws regarding child sexual abuse, (40) List of songs about child abuse, (41) Lloyd deMause, (42) Mandated reporter, (43) Mandatory reporting in the United States, (44) Masculism, (45) Mothers’ rights, (46) Multisystemic therapy, (47) National Center on Child Abuse and Neglect, (48) National Child Abuse Prevention Month, (49) National Society for the Prevention of Cruelty to Children, (50) Othermother, (51) Parental investment, (52) Parenting styles, (53) Parenting, (54) Paternal bond, (55) Royal Commission into Institutional Responses to Child Sexual Abuse, (56) Save the Children International, (57) Theraplay, (58) Trauma model of mental disorders, (59) Vicarious liability, (60) WAVE Trust, (61) Youth studies |
