# Supplementary material for: A Study on User-Oriented Subjects of Child Abuse on Wikipedia: Temporal Analysis of Wikipedia History Versions and Traffic Data
Source: J Med Internet Res. 2023 Jul 17;25:e43901. doi: 10.2196/43901 (PMC10390980; doi:10.2196/43901)
Supplement: Multimedia Appendix 2 [file jmir_v25i1e43901_app2.doc]

## **Articles created in the investigated 4 periods for each facet**

| Facets | Period | New articles |
| --- | --- | --- |
| Maltreatment behavior | 2010-2011 | Child sexual abuse in New York City religious institutions  Collingswood Boys  Franklin child prostitution ring allegations  Narcissistic abuse  Outline of domestic violence  Penn State child sex abuse scandal  Power and Control: Domestic Violence in America  Child sexual abuse accommodation syndrome  Destabilisation  Disability abuse  Institutional abuse  Sexual abuse scandal in the English Benedictine Congregation  Social undermining  Victim playing |
|  | 2012-2013 | Abusive power and control  Adverse Childhood Experiences Study  Derby child sex abuse ring  Oxford child sex abuse ring  Rotherham child sexual exploitation scandal  Telford child sex abuse ring  Child abuse (skin signs)  Death of Daniel Valerio  Jimmy Savile sexual abuse scandal  Rochdale child sex abuse ring |
|  | 2014-2015 | Athletes and domestic violence  Aylesbury child sex abuse ring  Banbury child sex abuse ring  Kasur child sexual abuse scandal  Bristol child sex abuse ring  Child abuse in New Zealand  Child sexual abuse in Australia  Child sexual abuse in Nigeria  Child sexual abuse in the United Kingdom |
|  | 2016-2017 | Child abuse in China  USA Gymnastics sex abuse scandal  Flying monkeys (psychology)  Halifax child sex abuse ring  Isolation to facilitate abuse  Keighley child sex abuse ring  List of child abuse cases featuring long-term detention  Mormon abuse cases |
| People and environment | 2010-2011 | Nurture kinship  Work-Family Balance in the United States |
|  | 2012-2013 | Enmeshment  Attachment theory and psychology of religion  Fathers as attachment figures  Inequality within immigrant families (United States) |
|  | 2014-2015 | Child soldiers in Sierra Leone |
|  | 2016-2017 | - |
| Problems and risks | 2010-2011 | Developmental impact of child neglect in early childhood  Effects of domestic violence on children  Healthy narcissism  Traumatic bonding |
|  | 2012-2013 | Vulnerable adult |
|  | 2014-2015 | Externalizing disorders |
|  | 2016-2017 | Atlas personality |
| Protection and support | 2010-2011 | List of songs about child abuse  Child sexual abuse laws in the United States  International Society for the Prevention of Child Abuse and Neglect  Multisystemic therapy (MST) |
|  | 2012-2013 | Karly’s Law  Child sexual abuse laws in India  Royal Commission into Institutional Responses to Child Sexual Abuse |
|  | 2014-2015 | Bikers Against Child Abuse  Child Abuse Review  Mandatory reporting in the United States  Child abuse image content list  Independent Inquiry into Child Sexual Abuse |
|  | 2016-2017 | Child Abuse & Neglect  National Child Abuse Prevention Month |
