# Supplementary material for: A Study on User-Oriented Subjects of Child Abuse on Wikipedia: Temporal Analysis of Wikipedia History Versions and Traffic Data
Source: J Med Internet Res. 2023 Jul 17;25:e43901. doi: 10.2196/43901 (PMC10390980; doi:10.2196/43901)
Supplement: Multimedia Appendix 3 [file jmir_v25i1e43901_app3.doc]

## **SOM displays, clusters, high-frequency terms, and subjects of each facet**

This appendix demonstrates the SOM displays and the tables of high-frequency terms and phrases for each facet. The color bars on the right side of Figures S1 to S4 represent different values of the U-matrix. Lower value means higher similarity. The numbers stand for the articles and the corresponding article of each number is presented in Multimedia Appendix 1. Every rectangle or polygon represents a cluster and the numbers in the same rectangle/polygon represent the articles belonging to the same cluster. The numbers not included in any rectangle/polygon stand for the isolated articles which were not grouped to any clusters. The clusters with more than three articles were the large clusters in purple while those with three or less entries were the small clusters in red.

Tables S1 to S4 display the high-frequency terms and phrases, and the subjects discovered within each large cluster based on the terms/phrases. The high-frequency terms/phrases were extracted from the articles in each cluster by the n-gram approach. This study only extracted the 2-word, 3-word, and 4-word phrases from the entries in each cluster. The high-frequency terms and phrases are displayed in the second column of each table and the frequency of each term/phrase is included in the brackets following the term/phrase. Because of the limitation of length, these tables only include the top 15 high-frequency terms/phrases. The researcher proposed the subjects of each large cluster by examining the high-frequency terms and phrases of it.

(1) The *Maltreatment behavior* facet

Figure S1 presents the SOM display of the *Maltreatment behavior* facet (F1). Seven large clusters and three small clusters were generated according to the clustering criteria. Table S1 shows the high-frequency terms and phrases of the seven large clusters, as well as the subjects generated for them.


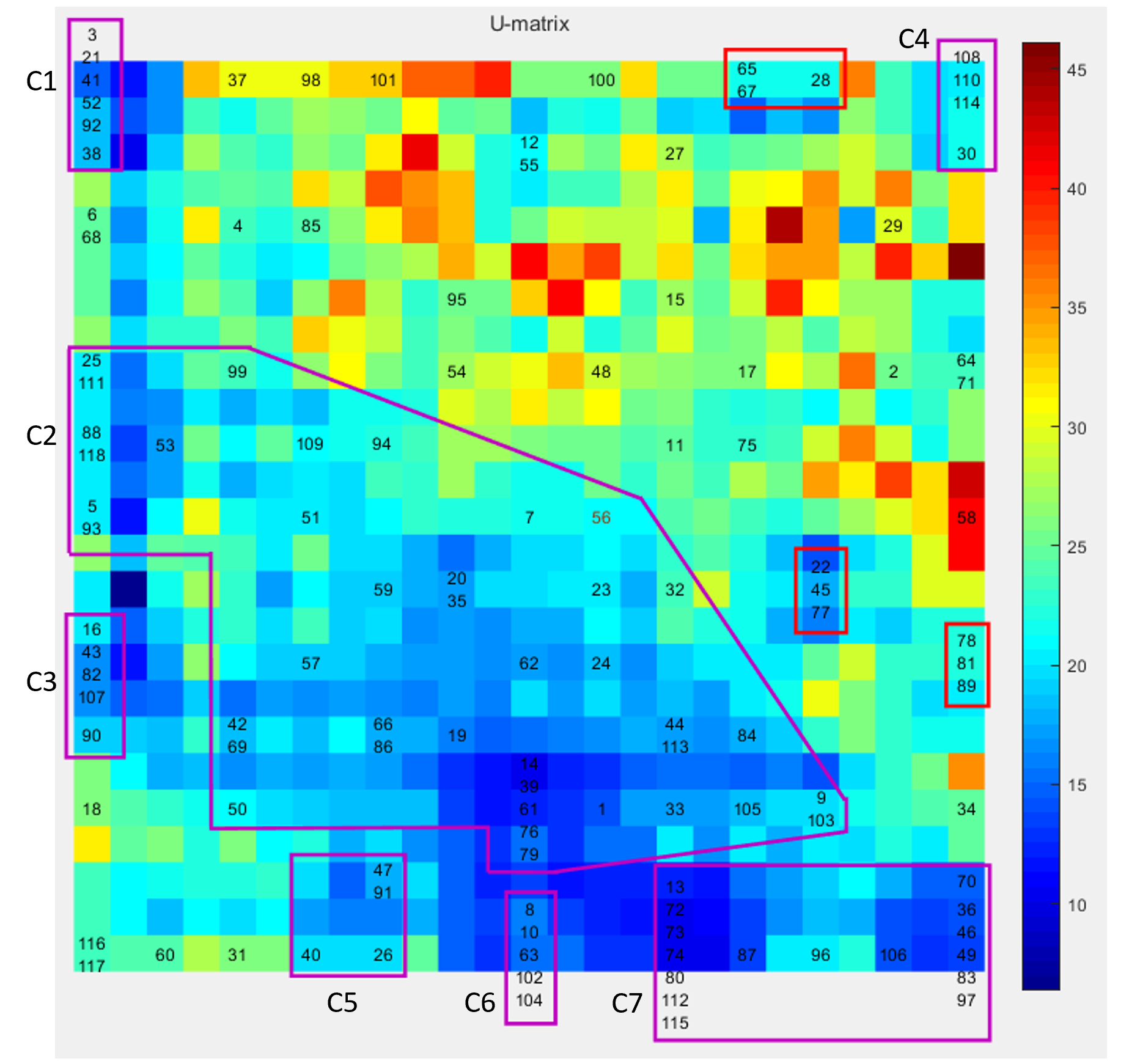


Figure S1. SOM Display of F1*.*

Table S1. Subjects of F1.

| Clusters | High-frequency terms and phrases | Subjects |
| --- | --- | --- |
| C1 | Domestic violence (282), child abuse (145), corporal punishment (123), violence against women (90), united states (88), human rights (70), intimate partner (67), partner violence (62), health organization (60), emotional abuse (56), intimate partner violence (55), World Health Organization (46), abuse and neglect (39), punishment of children (34), Council of Europe (26) | Emotional abuse, physical abuse, domestic violence, child abuse, neglect, violence against woman, health organization, government agencies and departments, victim, research on child abuse, disease control, disease prevention, reproduction, mental illness, physical illness, child marriage, health care, criminal justice |
| C2 | Sexual abuse (99), child abuse (94), social undermining (74), parental alienation (52), child neglect (45), child pornography (41), domestic violence (40), New York (36), mental health (34), social support (30), United States (24), child sexual abuse (19), the cruel mother (18), abuse and neglect (17), power and control (15) | Sexual abuse, child abuse, domestic violence, physical abuse, neglect, inequalities, social service, child abuse cases, school violence and bullying, research on child abuse, violence against woman, child pornography, health organization, mental illness, dysfunctional families, medical abuse, sexual exploitation, laws, therapies, child exploitation, emotional abuse, child and youth protection organization |
| C3 | Sexual abuse (54), child sexual abuse (20), narcissistic abuse (16), domestic violence (16), physical abuse (11), breaking the cycle (10), child abuse (8), cycle of violence (6), child-on-child sexual abuse (6), family violence (5), stress disorder (5), mental health (4), abused children (4), narcissistic supply (4), abuse neglect (4), Alice Miller (4) | Sexual abuse, domestic violence, physical abuse, emotional abuse, neglect, child and youth protection, mental illness |
| C4 | Sex tourism (61), slave trade (59), child sex tourism (48), united states (75), sexual exploitation (35), human trafficking (32), sexual slavery (30), forced labour (29), sex slaves (27), human rights (26), World War (25), New York (24), child pornography (22), unfree labour (19), sex trafficking (18) | Sexual abuse, sexual exploitation, child abuse, human trafficking, forced labor, news, marriage problem, law enforcement agency, survey and report, |
| C5 | Domestic violence (13), control domestic violence (8), Jill and Rob (7), Vanessa Jackson (6), power and control (5), attachment theory (4), Beth Thomas (4) | Domestic violence, news, mental illness |
| C6 | Sexual abuse (36), sexual activity (20), US Conference of Catholic (12), Child and Youth Protection (10), Fall River (9), child sex abuse (9), BBC News (7), Catholic diocese (6), abuse scandal (6), abuse cases (4), protection of children (4) | Sexual abuse, news, scandals, child abuse, child and youth abuse organization |
| C7 | Sexual abuse (118), Jimmy Savile (87), child abuse (82), sexual exploitation (65), BBC News (57), child sex abuse (55), abuse scandal (37), child sexual exploitation (36), Daily Telegraph (35), North Wales (35), sexual activity (35), child sexual abuse (27), South Yorkshire Police (19), child abuse scandal (16), child sex abuse ring (14) | Sexual abuse, child abuse, news, scandals, sexual exploitation, law enforcement agency, law enforcement agency, child and youth protection organization, social service, child exploitation, child pornography, government agencies and departments |

(2) The *People and environment* facet

According to Figure S2, the *People and environment* facet (F2) had two large clusters and three small clusters. The high-frequency terms and phrases, and the subjects of the large clusters are demonstrated in Table S2.


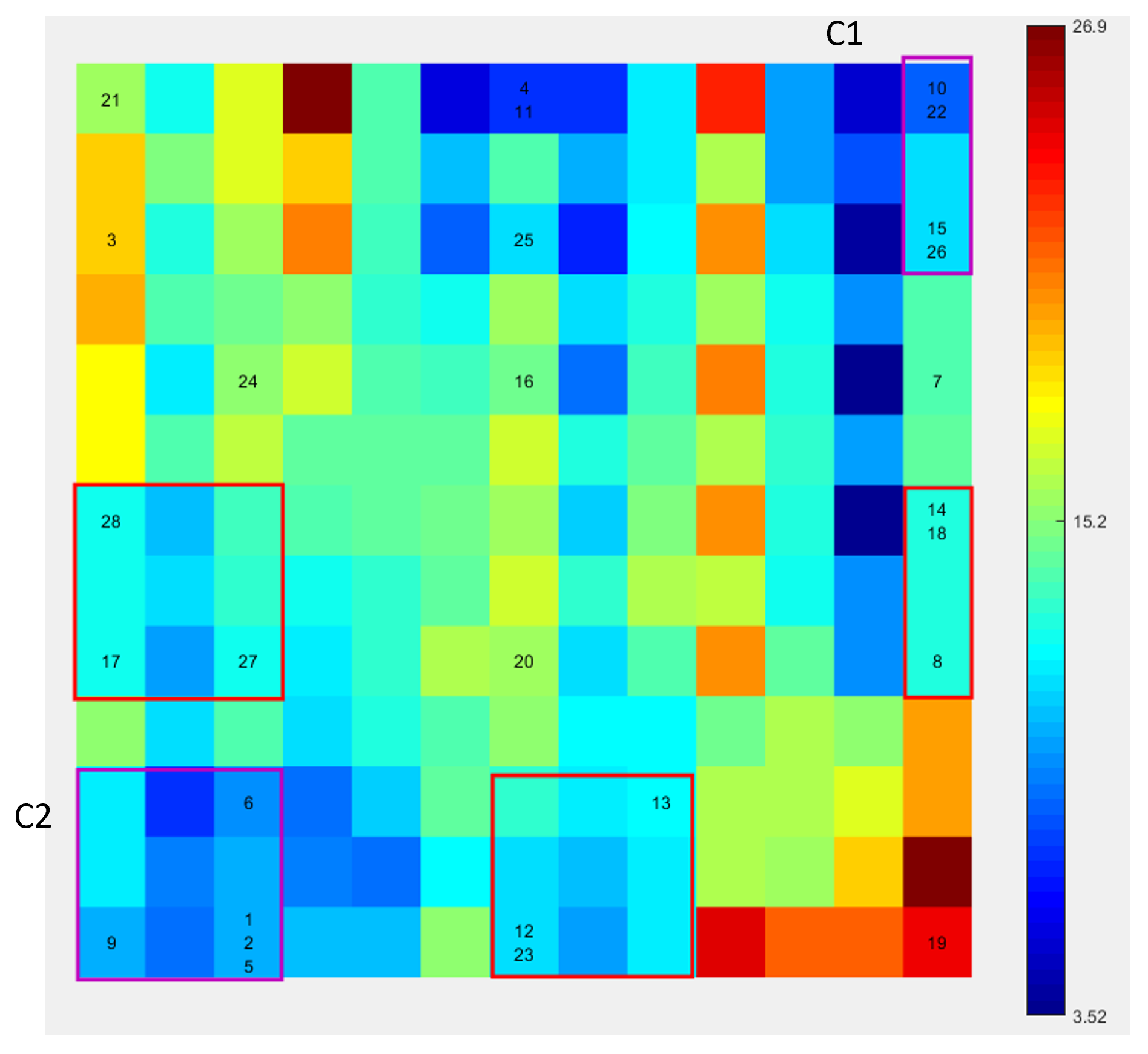


Figure S2. SOM Display of F2.

Table S2. Subjects of F2.

| Clusters | High-frequency terms and phrases | Subjects |
| --- | --- | --- |
| C1 | United States (63), family members (24), immigrant families (16), men and women (16), nuclear family (15), domestic violence (13), gender equality (12), family issues (12), marriage and family (12), middle class (11), legal status (11), child care (11), American culture (10), family life (10), lower class (10), Journal of Family Issues (10) | Immigrant family, nuclear family, domestic violence, inequalities, social class, child care, family policy, family model, marriage problem, minority, health care, child marriage |
| C2 | Attachment styles (101), working models (53), attachment theory (24), relational schemas (21), attachment figure (20), personal relationships (18), adult attachment (16), Social Psychology (15), Personality and Social Psychology (15), secure attachment (13), attachment system (9), romantic relationships (8), child development (8), changes in attachment styles (8), Mikulincer Shaver (7), marital satisfaction (7) | Reproduction, mental illness, child abuse |

(3) The *Problems and risks* facet

The *Problems and risks* facet (F3) had three clusters as shown in Figure S3 and the subjects of each cluster are presented in Table S3.


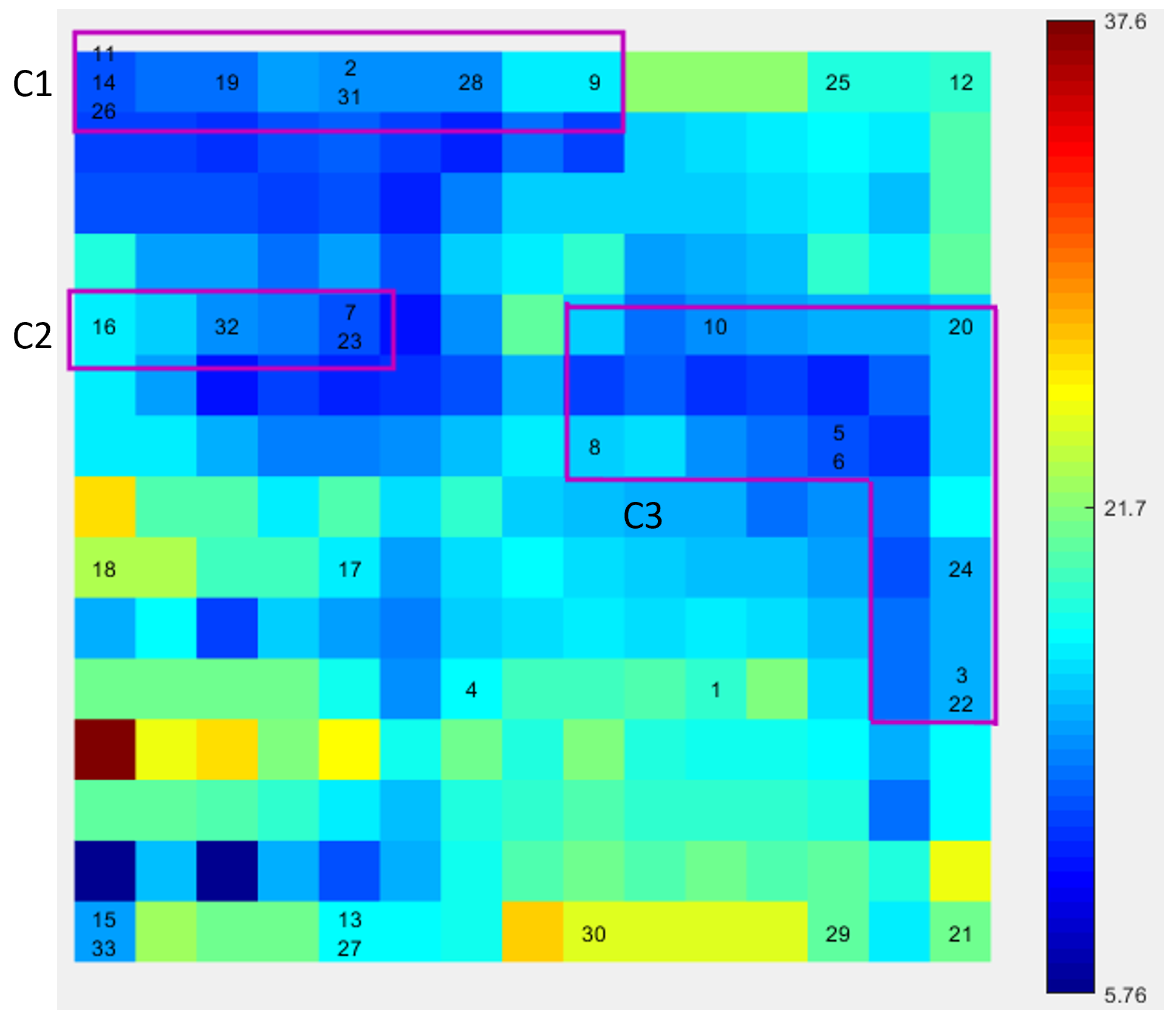


Figure S3. SOM Display of F3.

Table S3. Subjects of F3.

| Clusters | High-frequency terms and phrases | Subjects |
| --- | --- | --- |
| C1 | Emotional dysregulation (12), emotional regulation (8), mental health (10), personality disorder (7), traumatic bonding (6), narcissistic parents (6), self-psychology (6), healthy narcissism (5), Geschwind syndrome (5), early childhood (5), spiritual crisis (5), bipolar disorder (4), borderline personality (4), spiritual emergency (4), narcissistic parenting (4), posttraumatic stress (4), narcissistic personality disorder (4), temporal lobe epilepsy (4) | Mental illness |
| C2 | Vulnerable adult (23), neglected children (12), psychosomatic medicine (9), transactional analysis (8), child neglect (6), child abuse (5), psychosomatic disorders (4), family systems (4), Murray Bowen (4), stress disorder (4), posttraumatic stress (4), Karpman drama triangle (4), posttraumatic stress disorder (4), child abuse and neglect (4) | Mental illness, research on treatment and therapies, child abuse, neglect, emotional abuse |
| C3 | Conduct disorder (100), borderline personality disorder (94), people with BPD (61), domestic violence (56), attachment disorder (52), emotion regulation (46), mental health (34), reactive attachment (32), reactive attachment disorder (30), traumatic stress (23), American Psychiatric Association (18), child and adolescent (18), antisocial personality (17), oppositional defiant disorder (17), posttraumatic stress disorder (15) | Mental illness, domestic violence, research on treatment and therapies, therapies, child abuse, medical abuse, physical abuse, child abuse and youth protection program, |

(4) The *Protection and support* facet

Four large clusters and four small clusters were generated for the *Protection* *and support* facet (F4) as shown in Figure S4 and Table S4 displays the subjects of the large clusters.


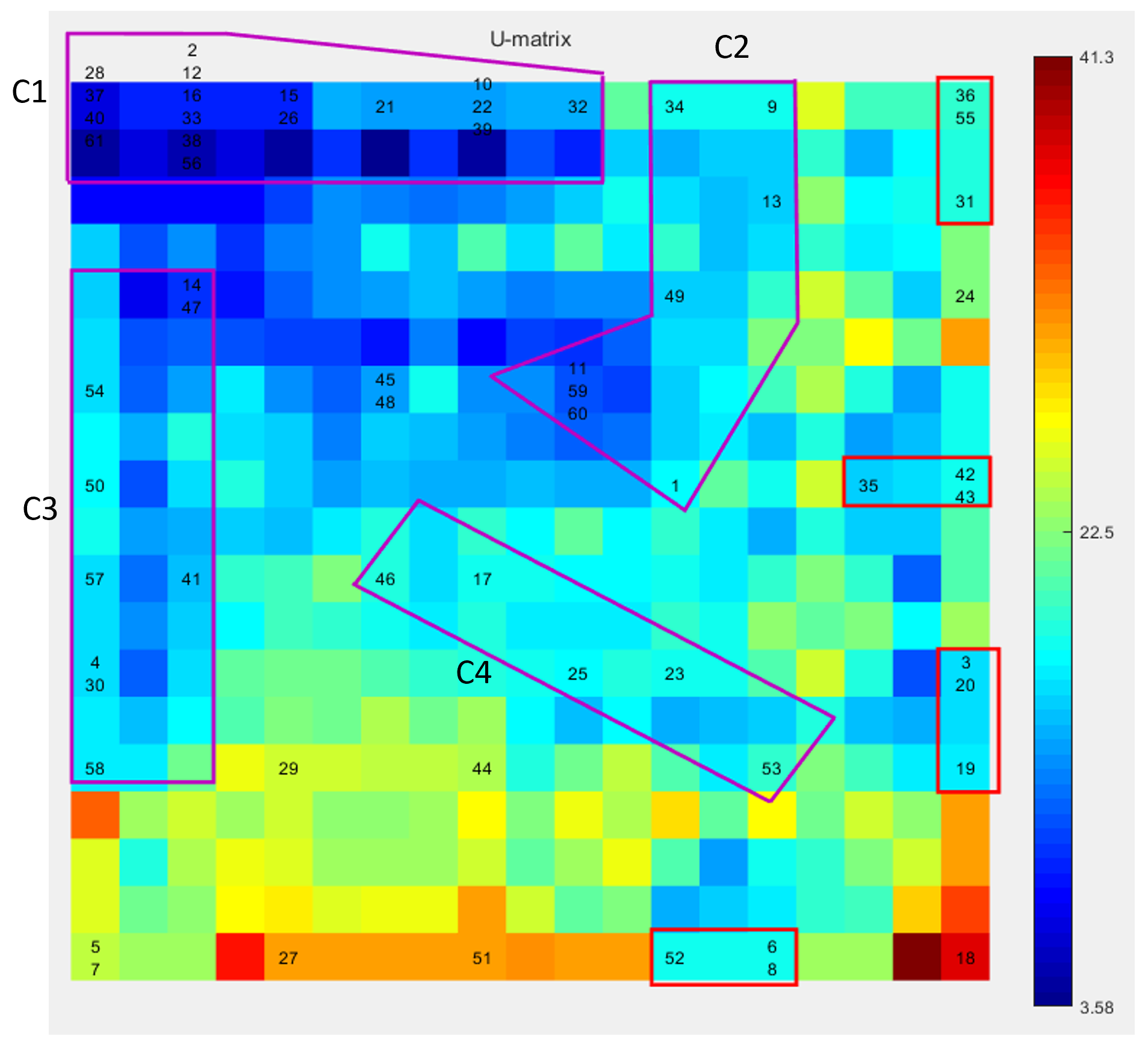


Figure S4. SOM Display of F4.

Table S4. Subjects of F4.

| Clusters | High-frequency terms and phrases | Subjects |
| --- | --- | --- |
| C1 | Child abuse (57), child sexual abuse (29), human rights (28), child abuse and neglect (16), youth studies (15), Public Law (14), protection of children (13), Abuse Prevention and Treatment (12), Sexual Offences Act (11), Child Abuse Prevention (9), abuse laws (8), Mountain Goats (8), Save the Children (8), sexual abuse laws (8), Prevention and Treatment Act (8) | Child abuse, sexual abuse, neglect, laws, child and youth protection organization, research on child abuse, government agencies and departments, government programs |
| C2 | Corporal punishment (39), Prevention of Cruelty (23), Cruelty to Children (14), abuse excuse (11), abuse defense (8), vicarious liability (8), child protection (8), the Guardian (7), United States (7), National Society (7), content blocking (6), Supreme Court (6), ritual abuse (6), young people (5), Irish Society (5), social services (5), United Nations (5), criminal law (5), Internet Watch Foundation (5) | Physical abuse, child abuse, laws, government agencies and departments, government programs , Judicial institutions, ritual abuse, social service, child and youth protection organization, child pornography |
| C3 | Child abuse (16), identified patient (8), mental health (7), Lloyd deMause (7), sexual abuse (7), mental disorders (7), Journal of Psychohistory (7), trauma model (6), attachment theory (6), black women (6), child abuse investigation (6), traumatic experiences (5), personality disorder (4), attachment disorders (4), mental health professionals (4), Health and Human (4) | Child abuse, sexual abuse, mental illness, research on treatment and therapy, child and youth protection organization, minority, health organization |
| C4 | Children’s rights (75), human rights (28), false allegations (19), child sexual abuse (13), United Nations (12), child development (11), United States (10), child abuse (9), child wellbeing (9), parenting style (8), multisystemic therapy (8), mental health (7), health care (7), child rearing (6), child mortality (5), physical integrity (5), youth rights (5), American communities (5), parenting styles (5), mortality rate (5), child deprivation (5), Child Development Index (5), Council of Europe (5) | Child and youth protection organization, criminal justice, child abuse, sexual abuse, parenting, therapies, health care, research on child abuse, government agencies and departments, physical abuse, Judicial institutions, minority |
