# Supplementary material for: A Study on User-Oriented Subjects of Child Abuse on Wikipedia: Temporal Analysis of Wikipedia History Versions and Traffic Data
Source: J Med Internet Res. 2023 Jul 17;25:e43901. doi: 10.2196/43901 (PMC10390980; doi:10.2196/43901)
Supplement: Multimedia Appendix 4 [file jmir_v25i1e43901_app4.docx]

## **Themes and subjects of each facet**

This appendix demonstrates the themes and subjects identified for each facet and cluster based on the high-frequency terms and phrases. Every facet is related to several themes and every theme is related to several facets. Every theme includes several subjects while every subject is included in only one theme.

| Facets | Themes | Subjects |
| --- | --- | --- |
| Maltreatment behavior  People and environment  Problems and risks  Protection and support | Abuse and violence | Child abuse  Community violence  Domestic violence  Emotional abuse  Medical abuse  Neglect  Physical abuse  Ritual abuse  School violence and bullying  Sexual abuse  Violence against woman |
| Maltreatment behavior | Child abuse cases | News  Victims  Scandals |
| Maltreatment behavior  People and environment  Problems and risks  Protection and support | Prevention of child abuse | Child and youth protection organization/program  Child care  Research on child abuse  Social service  Survey and report |
| Maltreatment behavior  Problems and risks  Protection and support | Treatment and therapies | Health care  Health organization  Health research on treatments and therapies  Therapies |
| Maltreatment behavior  Protection and support | Judicial and government administration | Criminal justice  Judicial institutions  Government agencies and departments  Government programs  Laws  Law enforcement agency |
| Maltreatment behavior  People and environment  Problems and risks  Protection and support | Health problems and diseases | Disease control  Disease prevention  Mental illness  Physical illness  Reproduction |
| Maltreatment behavior  Protection and support | Related social issues and crimes | Child exploitation  Child pornography  Discrimination  Forced prostitution  Human trafficking  Indecent assaults  Inequalities  Marriage problems  Minority  Sexual exploitation  Social class |
| Maltreatment behavior  People and environment  Protection and support | Related family issues | Dysfunctional families  Family policy  Family relations  Immigrant families  Nuclear family  Parenting |
