# Supplementary material for: A Study on User-Oriented Subjects of Child Abuse on Wikipedia: Temporal Analysis of Wikipedia History Versions and Traffic Data
Source: J Med Internet Res. 2023 Jul 17;25:e43901. doi: 10.2196/43901 (PMC10390980; doi:10.2196/43901)
Supplement: Multimedia Appendix 5 [file jmir_v25i1e43901_app5.doc]

## **Terms whose frequencies increased or decreased the most from 1 period to the next and the themes of these terms of each facet**

In this appendix, the changes of the themes in each theme from 1 period to another were explored. For each facet, the frequencies of its 2-,3-, and 4-word terms and phrases were counted and the frequency difference of each term/phrase from 1 period to next period was calculated. In other words, for each term/phrase, the difference of its frequency in period 2 and its frequency in period 1, the difference of periods 2 and 3, and the difference of periods 3 and 4 were all calculated. The terms/phrases of each facet were ranked according to their frequency differences and the terms/phrases whose frequencies increased or decreased the most from 1 period to next were extracted from the rankings. Tables S1 to S4 display the top 20 terms/phrases of the rankings and only the terms/phrases whose frequencies increased or decreased more than 4 are included in the tables. The themes relevant to the terms/phrases were also included in these tables. The numbers in each table show the frequency differences. If a term’s frequency decreased from periods 1 to 2, its frequency difference would be negative and vice versa.

(1) The *Maltreatment and behavior* facet (F1)

Table S1. Changes of themes in the four periods in F1.

| Period | High-frequency terms and phrases | | Themes |
| --- | --- | --- | --- |
| Period 1 VS. Period 2 | Frequency decreasing terms | Human trafficking (-135), South America (-12), Eastern Europe (-9), West Africa (-8), domestic violence (-7), Western Europe (-7), family violence (-6), male victims (-5) | Abuse and violence, related social issues and crimes |
|  | Frequency increasing terms | Sexual abuse (126), sex ratio (119), child abuse (110), BBC News (98), Penn State (89), sex abuse (87), social undermining (70), United States (68), child sexual abuse (66), sex trafficking (64), New York (59), child sex (59), child pornography (37), sexual exploitation (32), Daily Telegraph (32), abuse cases (29), human rights (29), sex-selective abortion (26), law enforcement (25), trafficking victims (24), social support (24) | Abuse and violence, prevention of child abuse, related social issues and crimes, health problems and diseases, judicial and government administration |
| Period 2 VS. Period 3 | Frequency decreasing terms | Catholic sexual abuse scandal (-9), social undermining (-7), trafficking victims (-6), Catholic sex abuse cases (-6), coercive persuasion (-6), forced labour (-6), New York Times (-5), Daily News (-5), high rates (-5), school corporal punishment (-5) | Abuse and violence, child abuse cases, related social issues and crimes |
|  | Frequency increasing terms | Domestic violence (136), sexual abuse (131), child abuse (85), corporal punishment (79), human rights (71), violence against women (65), United Nations (62), sexual exploitation (50), New York (48), BBC News (42), childhood experiences (41), partner violence (35), child sexual abuse (34), family violence (33), adverse childhood experiences (32), World Health Organization (31), sex ratio (28), mental health (26), punishment of children (26), parental alienation (26) | Abuse and violence, prevention of child abuse, judicial and government administration, related social issues and crimes, related family issues, health problems and diseases |
| Period 3 VS. Period 4 | Frequency decreasing terms | Corporal punishment (-18), Rochdale sex trafficking (-14), Taylor and Francis (-14), termination of pregnancy (-12), sex trafficking (-8), American Psychological Association (-7), abuse of children (-7), National Council on Family (-7), Council on Family Relations (-7), sex gang (-6), prisoners of war (-6), sex grooming (-6), human rights (-5), Global Initiative (-5), Daily Mail (-5), emotional abuse (-5), primary school (-5), intimate terrorism (-5), Child and Youth Protection (-5) | Abuse and violence, related social issues and crimes, child abuse cases, health problems and diseases, prevention of child abuse, related family issues |
|  | Frequency increasing terms | Sexual abuse (156), child sexual abuse (62), Penn State (35), sexual exploitation (33), United States (32), sexual activity (26), Bryn Alyn (25), sexual assault (23), indecent assault (20), BBC News (19), child maltreatment (19), violence against children (18), South Yorkshire Police (17), adverse childhood experiences (15), abuse scandal (15), child prostitution (15), child neglect (15), localised grooming (15), human trafficking (14), sex-selective abortion (14) | Abuse and violence, related social issues and crimes, prevention of child abuse, child abuse cases, health problems and diseases |

(2) The *People and environment* facet (F2)

Table S2. Changes of themes in the four periods in F2.

| Period | High-frequency terms and phrases | | Themes |
| --- | --- | --- | --- |
| Period 1 VS. Period 2 | Frequency decreasing terms | Oxford University (-11), antisocial behavior (-6), marriage and family (-6) | Health problems and diseases, related family issues |
|  | Frequency increasing terms | United States (52), family members (17), child support (16), Child Development (15), attachment figure (14), attachment security (13), extended family (11), domestic violence (8), nuclear family (6), men and women (6), attachment theory (5), family economics (5), individual differences (5) | Related family issues, prevention of child abuse, abuse and violence, treatment and therapies |
| Period 2 VS. Period 3 | Frequency decreasing terms | - | - |
|  | Frequency increasing terms | Men and women (17), United States (15), nuclear family (11), United Nations (11), domestic violence (10), family life (7), gender equality (7), gender roles (6), child abuse (5), nurture kinship (5), raising children (5), equal rights (5) | Related family issues, abuse and violence, related social issues and crimes, prevention of child abuse |
| Period 3 VS. Period 4 | Frequency decreasing terms | Maternity leave (-8), men and women (-5), working mothers (-5) | Related social issues and crimes |
|  | Frequency increasing terms | United States (10), marriage and family (8), father’s rights (8), New York (5), father’s rights movement (5) | Related social issues and crimes |

(3) The *Problems and risks* facet (F3)

Table S3. Changes of themes in the four periods in F3.

| Period | High-frequency terms and phrases | | Themes |
| --- | --- | --- | --- |
| Period 1 VS. Period 2 | Frequency decreasing terms | Disease Control and Prevention (-46), traumatic stress (-15), sexual abuse (-12), heart disease (-11), population health (-10), social determinants of health (-9), complex trauma (-7), Sudden Infant Death Syndrome (-7), foster children (-6), risk factors (-6), physical abuse (-6), van Der Kolk (-6), conduct problems (-5), clinical psychology (-5), foster parent (-5) | Health problems and diseases, abuse and violence, judicial and government administration |
|  | Frequency increasing terms | People with BPD (77), infant mortality (68), emotion regulation (42), personality disorder (33), borderline personality disorder(31), mortality rate (21), foster care (19), blunted affect (15), substance abuse (14), birth weight (12), conduct disorder (11), bipolar disorder (10), domestic violence (9), oppositional defiant disorder(9), substance use order(9), emotional regulation (9), drug abuse (8), psychiatric association (8), diagnostic criteria (8), personality disorders (8), flat affect (8), New York(8) | Health problems and diseases, abuse and violence |
| Period 2 VS. Period 3 | Frequency decreasing terms | Substance abuse (-9), oppositional defiant disorder (-6) | Health problems and diseases |
|  | Frequency increasing terms | Washington DC (15), infant mortality (14), child molesters (12), conduct problems (11), sudden infant death (11), personality disorder (10), antisocial behavior (10), Sudden Infant Death Syndrome (10), conduct disorder (9), mortality rate (8), antisocial personality (8), sexual abuse (8), traumatic stress (8), women’s health (7), assessment and treatment (7), chronic stress (7), determinants of health (7), borderline personality (6), Blanchard R (6), child pornography (6), sexual behavior (6) | Health problems and diseases, abuse and violence, treatment and therapies, related social issues and crimes |
| Period 3 VS. Period 4 | Frequency decreasing terms | Drug abuse (-6), Cantor JM (-6), van Der Kolk (-5), Blanchard R (-5) | Health problems and diseases |
|  | Frequency increasing terms | Women’s health (50), United States (35), health care (21), United Nations (21), developing countries (18), World Health Organization (17), developed countries (16), substance use disorders (14), infant mortality (13), mental health (13), New York (12), borderline personality (12), public health (10), mortality rate (9), Child Health and Human Development (8), family members (8), substance abuse (8), health issues (7), disease control (7), personality disorder (6) | Health problems and diseases, treatment and therapies, prevention of child abuse, abuse and violence, related family issues |

(4) The *Protection and support* facet (F4)

Table S4. Changes of themes in the four periods in F4.

| Period | High-frequency terms and phrases | | Themes |
| --- | --- | --- | --- |
| Period 1 VS. Period 2 | Frequency decreasing terms | Family members (-7), Jehovah’s witnesses (-6), child wellbeing (-6), attachment parenting (-5), parenting style (-5) | Related family issues, treatment and therapies |
|  | Frequency increasing terms | Child abuse (50), sexual abuse (37), Amber Alert (27), child sexual abuse (20), cognitive development (17), child protection (16), Watch Tower Society (14), Child Abuse and Neglect (14), Department of Health (11), Haut de la Garenne (9), Catholic Church (8), United States (7), Supreme Court (7), Health and Human Services (7), marriage and family (7), family therapy (6), parental investment (6), child development (5), social work (5), United Kingdom (5), alert system (5) | Abuse and violence, child abuse cases, prevention of child abuse, judicial and government administration, related social issues and crimes |
| Period 2 VS. Period 3 | Frequency decreasing terms | Family therapy (-18), attachment theory (-12), bodies of elders (-9), mandatory reporting (-9), substance abuse (-8), family members (-8), Journal of Family (-7), Juvenile Justice (-6), Cassidy J (-6), Developmental Psychology (-5), attachment theory research (-5) | Treatment and therapies, health problems and diseases, prevention of child abuse, abuse and violence, judicial and government administration |
|  | Frequency increasing terms | Sexual abuse (139), child sexual abuse (101), Commission into Institutional Responses (51), child abuse (40), Amber Alert (27), Royal Commission (27), corporal punishment (25), child protection (21), Children Act (20), attachment figure (15), Jehovah’s witnesses (11), parenting styles (10), child’s needs (10), Save the Children (10), child maltreatment (9), missing children (9), Human Development (8), Blehar M (8), human rights (8), parenting style (8), New South Wales (8) | Abuse and violence, judicial and government administration, prevention of child abuse, related family issues |
| Period 3 VS. Period 4 | Frequency decreasing terms | Save the Children (-11), Force Report (-10), attachment style (-6) | Prevention of child abuse, treatment and therapies |
|  | Frequency increasing terms | Attachment parenting (143), sexual abuse (93), child sexual abuse (73), child abuse (58), Commission into Institutional Responses (53), New York (31), Royal Commission (27), parental investment (26), mandatory reporting (20), Child Abuse Prevention (17), child protection (16), Child Abuse and Neglect (16), United States (16), Catholic church (13), abuse inquiry (11), case study (11), foster care (10), child maltreatment (9), child care (9), sexual selection (9) | Related family issues, abuse and violence, judicial and government administration, prevention of child abuse, related social issues and crimes |
