# Supplementary material for: A Study on User-Oriented Subjects of Child Abuse on Wikipedia: Temporal Analysis of Wikipedia History Versions and Traffic Data
Source: J Med Internet Res. 2023 Jul 17;25:e43901. doi: 10.2196/43901 (PMC10390980; doi:10.2196/43901)
Supplement: Multimedia Appendix 6 [file jmir_v25i1e43901_app6.doc]

## **The User-Oriented Subject Schema of Child Abuse on Wikipedia**

| Topic | Facets | Themes | Subjects | |
| --- | --- | --- | --- | --- |
| Child abuse | Maltreatment behavior  People and environment  Problems and risks  Protection and support | Abuse and violence | Child abuse  Domestic violence  Medical abuse  Physical abuse  School violence and bullying  Violence against woman | Community violence  Emotional abuse  Neglect  Ritual abuse  Sexual abuse |
|  |  | Child abuse cases | News  Scandals | Victims |
|  |  | Prevention of child abuse | Child and youth protection organization/program  Research on child abuse | Child care  Social service  Survey and report |
|  |  | Treatment and therapies | Health care  Health research on treatments and therapies | Health organization  Therapies |
|  |  | Judicial and government administration | Criminal justice  Government agencies and departments  Laws | Judicial institutions  Government programs  Law enforcement agency |
|  |  | Health problems and diseases | Disease control  Mental illness  Reproduction | Disease prevention  Physical illness |
|  |  | Related social issues and crimes | Child exploitation  Discrimination  Human trafficking  Inequalities  Minority  Social class | Child pornography  Forced prostitution  Indecent assaults  Marriage problems  Sexual exploitation |
|  |  | Related family issues | Dysfunctional families  Family relations  Nuclear family | Family policy  Immigrant families  Parenting |
